# Non-pharmacological interventions and corticosteroid injections for the management of the Achilles tendon in inflammatory arthritis: a systematic review

**DOI:** 10.1186/s13047-021-00484-6

**Published:** 2021-07-10

**Authors:** Shaily Modi, Deborah Turner, Kym Hennessy

**Affiliations:** 1 Western Sydney Local Health District, NSW Health, PO Box 574, Wentworthville, NSW 2145 Australia; 2 School of Clinical Sciences, Queensland University of Technology, GPO Box 2434, Brisbane, QLD 4001 Australia; 3 School of Health Sciences, Western Sydney University, Locked Bag 1797, Penrith, NSW 2751 Australia

**Keywords:** Achilles tendon, Inflammatory arthritis, Corticosteroid injection, Non-pharmacological interventions, Ultrasound

## Abstract

**Background:**

Achilles tendon (AT) pathologies, particularly Achilles enthesitis, are common in inflammatory arthritis (IA). Although there are various non-pharmacological interventions and injection therapies available, it is unknown if these interventions are effective for people with IA, as this population is often excluded from studies investigating the management of AT pathologies. This study aimed to identify and critically appraise the evidence for non-pharmacological interventions and corticosteroid injections in the management of AT pathology in those with IA.

**Methods:**

All studies which met the inclusion criteria (AT interventions in adults with a working clinical diagnosis of IA, English language) were identified from the following databases: Medline, Embase, CINAHL and the Cochrane Library. The search strategies used the search terms ‘spondyloarthropathies’, ‘inflammatory arthritis’, ‘achilles tendon’, ‘physical therapy’, ‘conservative management’, ‘injections’, and related synonyms. Studies included were quantitative longitudinal design, such as randomised controlled trials, pseudo randomised and non-randomised experimental studies, observational studies, cohort studies, and case control studies. All outcome measures were investigated, quality assessment to determine internal and external validity of included studies was undertaken, and qualitative data synthesis was conducted.

**Results:**

Of the 10,911 articles identified in the search strategy, only two studies that investigated the efficacy of corticosteroid injections for the management of the AT in IA met the inclusion criteria, and no studies were identified for non-pharmacological interventions. Both injection studies had low quality rating for internal and external validity, and thus overall validity. The included studies only investigated two outcome domains: pain and ultrasound (US) (B Mode and Doppler) identified abnormalities and vascularity in the AT. There is weak evidence suggesting a short-term improvement (6–12 weeks) in pain and for the reduction in some abnormal US (B-Mode and Doppler) detectable features (entheseal thickness, bursitis, and entheseal vascularity) at the AT and surrounding structures post-corticosteroid injection.

**Conclusion:**

Weak evidence is available regarding the efficacy of corticosteroid injections in reducing pain and inconclusive evidence for the improvement of abnormal US detectable features. No studies were identified for non-pharmacological interventions. It is evident from the lack of relevant literature that there is an urgent need for more studies assessing non-pharmacological interventions for the AT in people with IA.

**Supplementary Information:**

The online version contains supplementary material available at 10.1186/s13047-021-00484-6.

## Background

Inflammatory Arthritis (IA) refers to a group of conditions including rheumatoid arthritis (RA), ankylosing spondylitis (AS), psoriatic arthritis (PsA), and other spondyloarthropathies (SpAs) [1]. These conditions are progressive and characterised by joint destruction, pain, and eventually lead to decreased function [2].

IA has been shown to have a profound impact at both an individual level (negative impact on quality of life of those afflicted) and at a societal level (medical expenditure and work disability) [1–3]. Enthesitis (inflammation occurring at the attachment site of tendons and ligaments to bone) is regarded to be a hallmark feature of the SpAs [3] and a known predilection to the insertion of the Achilles tendon has been reported in the literature [4]. The prevalence of Achilles enthesitis in IA, in particular SpAs, is much higher than the general population [5, 6]. Anecdotal evidence suggests that enthesitis in the SpAs may be largely unresponsive to standard pharmacological regimens, with treatment guidelines not recommending conventional disease-modifying anti-rheumatic drugs (DMARDs) due to reported failed efficacy on peripheral enthesitis [7]. The use of biological drug therapies has been described in the literature for those with NSAID-refractory persistent heel enthesitis [8]. However, there is evidence of the progression of pain and disability even when low disease activity has been achieved with the use of biological drug therapies [9].

An association between enthesitis, and the presence of higher disease activity, increased fatigue, worse functional status, reduced disease duration, and body mass index was reported in patients with AS [10]. In contrast to the accepted view that general chronic AT pathologies are primarily degenerative in nature in non IA populations, the involvement of a greater inflammatory component in those with IA has been acknowledged [11]. This highlights the need for targeted therapies to address both inflammatory and biomechanical features affecting the AT, as proposed by Woodburn et al. [12] for management of rheumatoid arthritis in patients with low, moderate and high disease activity.

Currently, high quality reviews that can be applied in clinical practice for the management of AT are unavailable. The available Cochrane review by MacLauchlan and Handoll [13] was withdrawn from the Cochrane Database due to lack of recency and the need for updating, and the Cochrane review protocol developed by Wilson et al. [14] in 2011 was subsequently withdrawn due to lack of progress. Recent systematic reviews proposing management options for the AT have been published, and include many conservative interventions and injection therapies [15–17]. Physical therapies, such as progressive heavy load eccentric exercises, gained popularity from the Alfredson’s protocol, and have shown promising efficacy in reducing pain in recent literature [16, 18]. Extracorpeal shockwave therapy (ESWT) has also been reported as being as effective as eccentric exercises [16]. Other conservative management options include orthoses, splints, low level laser therapy, and microcurrent therapy. However, these have not been proven to be as effective as eccentric exercises in studies [15, 16, 19]. Corticosteroids injections are powerful anti–inflammatories, and can be useful in patients that have previously experienced adverse reactions to non-steroidal anti-inflammatory drugs (NSAIDs) [20]. These are injected locally at the AT to decrease inflammation [17]. Although the mechanism through which corticosteroids have an effect is unknown, there is evidence to suggest corticosteroids can be effective in chronic tendinopathy at relieving pain, reducing swelling and inflammation [11, 17]. However, a risk of potential adverse effects, such as local infection, bleeding, swelling, and tendon rupture, is associated with corticosteroid injections. The potential adverse effects of the tendon itself are local bleeding and weakening of the tendon [17].

Although enthesitis and inflammatory AT pathologies are a hallmark of IA, those with IA are excluded from studies focused on the management of AT pathologies. Consequently, it is currently unknown if treatments for AT pathologies are safe and effective for people with IA. This is an important consideration as there is increased risk of adverse events due to immunosuppression and studies have shown different pathological processes at the AT for people with and without IA. Therefore, the aim of this review was to identify and critically appraise the evidence for the effectiveness of non-pharmacological interventions and corticosteroid injections for the management of the AT in people with IA.

## Methods

### Search strategy

A detailed electronic database search of the literature was performed on August 3rd, 2020 using the following databases: Medline, Embase, CINAHL, and the Cochrane Library. Reference lists of eligible studies were hand searched by the reviewers (SM and KH) to identify any relevant studies that were not identified through the initial electronic database search. The following search terms ‘spondyloarthropathies’, ‘inflammatory arthritis’, ‘achilles tendon’, ‘physical therapy’, ‘conservative management’, ‘injections’, and related synonyms were used to develop a comprehensive pragmatic literature search strategy. Standard MeSH or medical headings and appropriate keywords were utilised where appropriate according to each database. Boolean operators (such as ‘OR’, “AND’, and ‘*’) were used as appropriate. The full search strategies are available in Additional File 1.

### Study inclusion criteria

Studies included were quantitative longitudinal study designs, such as randomised controlled trials (RCTs), pseudo randomised and non-randomised experimental studies, cohort studies, and case control studies. Single case studies were excluded. Full text studies published only in the English language were included due to language barriers of the independent reviewers. There were no limitations on the year of publication. Studies reporting participants aged ≥18 years with a working diagnosis of IA made by a rheumatologist, and AT pathology were included. Studies investigating non-pharmacological interventions (such as orthoses and physical therapy) and/or site-specific corticosteroid injections were selected for further analysis. Limitations were not placed on which qualified health professional prescribed and/or administered the non-pharmacological interventions or corticosteroid injections at the AT. All outcome measures were selected for further analysis. Studies were excluded if they were not an intervention study or focused solely on pharmacological treatments, such as biologic drugs as a primary treatment.

### Study selection

All titles and abstracts of the studies found electronically and through reference list reviews were cross-referenced, and any duplicates were removed. Two reviewers (SM and KH) independently screened the title and abstracts of the studies for information fulfilling the eligibility criteria as described above. Any discrepancies in opinions were resolved through discussion. Full text articles were retrieved from the selected abstracts and compared to the inclusion criteria. Two reviewers (SM and KH) independently screened the full text articles to determine if they met the inclusion criteria. Any discrepancies were resolved through discussion.

### Quality assessment

The quality of the studies was assessed using criteria that assessed the internal validity (i.e. how well the study was conducted, including method errors or risk of bias) and external validity (i.e. generalisability of results to the wider population of people with IA and affected AT). The quality assessment criteria used was adapted from the Cochrane Collaboration tool [21] as previously reported by Hennessy et al. [22]. It included internal validity criteria of sequence generation and allocation concealment, blinding of participants, personnel and outcome assessors, incomplete outcome data, selective reporting and statistical issues, and interventions. External validity criterion assessed the representativeness of the sample population to the general population with IA and affected AT and the restrictiveness of the inclusion and exclusion criteria [21, 22]. Quality assessment was conducted by two independent reviewers (SM and KH). Any disagreements between the two reviewers were resolved through discussion. For a high quality rating, all applicable domains had to be scored as high quality. Any study with ≥1 domain scoring low quality resulted in an overall low quality rating for the study. This quality rating was agreed upon by the co-authors a priori, and has been used in previous systematic reviews [22, 23].

### Data extraction/ evidence grading

For outcome measures, random-effects model meta-analyses would have been conducted if multiple RCTs had been available. As multiple RCTs were not available, qualitative data synthesis was conducted. An evidence rating was assigned to the extracted data that was analysed and synthesised. Once the studies were rated for quality, they were grouped according to intervention and associated outcome measures, and evidence rating were assigned according to the criteria adapted from Ariens et al. [24] (Table 1). The interpretations of findings were based on combining the qualitative data synthesis and the evidence grading system.

**Table 1:** - Evidence Rating Criteria [24]

Level	Criteria
Strong	At least 2 studies of high quality with consistent findings (agreement of > 75% of studies)
Moderate	1 high quality study and at least 2 low-quality studies with consistent findings (agreement of > 75% of studies)
Weak	At least 2 low-quality studies with consistent findings (agreement of > 75% of studies)
Inconclusive	Insufficient and/or conflicting studies

## Results

**Fig. 1:**
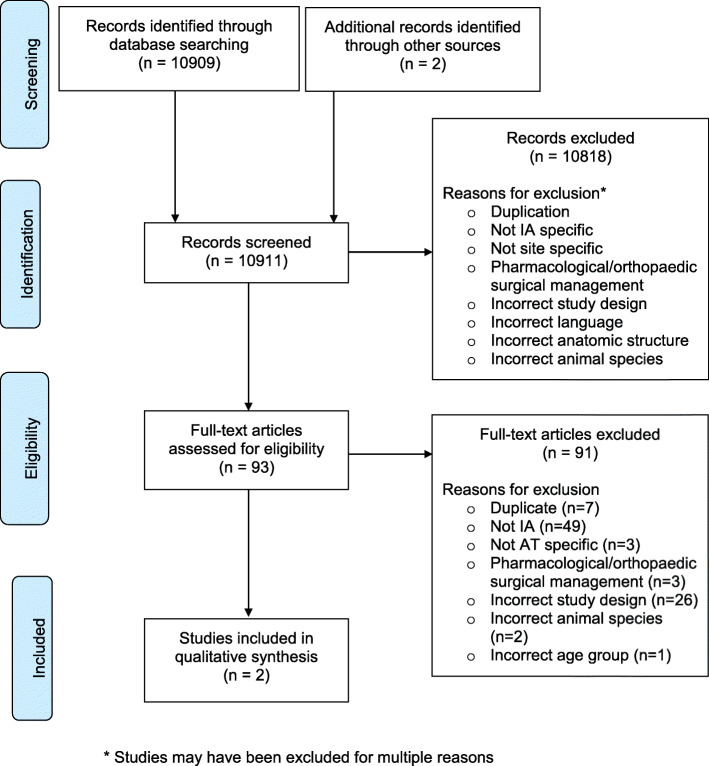
Search flowchart for non-pharmacological interventions and corticosteroid injections for the Achilles tendon in inflammatory arthritis

Using the detailed search strategy, a total of 10,909 articles were retrieved from Medline, Embase, CINAHL and Cochrane Library. Two articles were also identified through reference list reviews. Figure 1 outlines the flowchart displaying how the relevant studies were found [25]. The inclusion criteria were met by two studies (described in Table 2), which were both observational studies that assessed the effects of injecting corticosteroid around the site of the AT [26, 27]. No studies assessing non-pharmacological interventions (such as orthoses and physical therapy) for the AT in patients with IA were found.

**Table 2:** – Description of Included Studies

Author, year	Study Type	Participant Description	No. Entered / Completed study	Follow up period	Intervention	Outcome measures
**Huang et al.** [26]	OBS	AS patients with unilateral refractory AT enthesitis • M:F 6:1 • Disease Duration: 6.1 ± 5.7 years)	12 entered/ 12 completed (12 AT). 7 treated with corticosteroid	2,4,8 and 12 weeks	7 patients injected with 1 ml betamethasone	o Pain o Entheseal thickness (US derived) o Bone erosion o Bursitis o Enthesophyte
**Srivastava & Aggarwal** [27]	OBS	SpA patients with symptomatic AT enthesitis • M:F 8:1 • Subtypes: AS–10 JSpA–5 PsA–1 IBDA–1 USpA-1	29 patients (40 AT) entered/ 18 patients (27 AT) completed	6 weeks	20 mg methylprednisolone injected	o Pain o Entheseal thickness o Bone erosion o Bursitis o Enthesophyte o Entheseal hypoechogenecity (Doppler US) o Peritendinous oedema

*OBS* Observational study, *AT* Achilles tendon, *US* Ultrasound, *M:F* Male:Female ratio,

*AS* Ankylosing spondylitis, *SpA* Spondyloarthropathies, *JSpA* Juvenile Spondyloarthropathy, *PsA* Psoriatic Arthritis,

*IBDA* Inflammatory Bowel Disease-associated Arthritis, *USpA* Undifferentiated Spondyloarthropathy.

The study by Huang et al. [26] compared the efficacy of ultrasound (US) guided injections of etanercept and betamethasone (corticosteroid) when injected into the entheses of patients with AS and refractory Achilles enthesitis. The study by Srivastava & Aggarwal [27] investigated the efficacy of US guided corticosteroid (methylprednisolone) injections at Achilles enthesis in patients with SpAs. In the case of Huang et al. [26], only the corticosteroid injection reported outcomes for the seven patients who were injected with betamethasone were extracted, as the inclusion criteria of our systematic review did not include biologic pharmacological agents such as etanercept.

The sample size in both studies were small, lacked a robust sample size calculation, and had a male gender predominance (M:F = 6:1 [26], M:F = 8:1 [27]), thus making it hard to deduce any potential gender specific differences in results. Heterogeneity in the type of IA existed across the two studies, with Huang et al. [26] solely including patients with AS, whereas Srivastava & Aggarwal [27] represented subcategories of SpA, including those diagnosed with AS, juvenile SpA, PsA, inflammatory bowel disease-associated arthritis, and undifferentiated SpA.

The two studies were rated as having overall low internal and external validity during quality assessment (Table 3). Two outcome domains were identified: pain and US (B Mode and Doppler) identified abnormalities and vascularity in the AT. Considerable variation in the study duration and timing of outcomes assessments was noted in the two studies. Huang et al. [26] measured clinical parameters at baseline with follow up outcomes measures reported at 2, 4, 8 and 12 weeks. Srivastava & Aggarwal [27] had a shorter follow-up duration with the assessment of clinical parameters only at baseline and 6 weeks. Additionally, Huang et al. [26] investigated patients that had unilateral AT enthesitis (only one foot investigated), whereas Srivastava & Aggarwal [27] investigated symptomatic AT, which meant an individual participant may have had both ATs assessed if they were symptomatic. The loss to follow up varied across the studies, with no loss to follow up reported by Huang et al. [26], but considerable attrition (38% of study participants) was reported by Srivastava & Aggarwal [27], with a failure of the authors to offer any explanatory cause for the high levels of attrition in the study.

**Table 3:** - Quality Assessment of Included Studies

Author, year	Sequence Generation / Allocation Concealment (internal validity)	Blinding of Participants, Personnel, and Outcome Assessors (internal validity)	Incomplete Outcome Data (internal validity)	Selective Outcome Reporting / Statistical Issues (internal validity)	Interventions (internal validity)	Generalisability (external validity)	Internal Validity	External Validity
**Huang et al.** [26]	N/A	N/A	Yes	No	No	No	**Low**	**Low**
**Srivastava & Aggarwal** [27]	N/A	N/A	Yes	No	No	No	**Low**	**Low**

● Same checklist used for all study designs but N/A for domains 1 and 2 for study designs other than RCT

● *N/A* Not applicable

**3 domains for Internal Validity**

YES for all of the domains = HIGH QUALITY

NO for any domain = LOW QUALITY

**1 domain for External Validity**

YES for domain = HIGH QUALITY

NO for domain = LOW QUALITY

Due to the lack of RCTs and/or sufficient studies/data for analysis, meta-analyses could not be conducted, as was the original intention of this work. A qualitative synthesis of the results is outlined below and described in Table 4.

**Table 4:** – Qualitative synthesis of results and overview of evidence

	DOMAIN								
	Pain	Ultrasound Identified Abnormalities and Vascularity							
		B Mode Ultrasound						Doppler Ultrasound	
	Visual Analogue Scale (units not specified)	Entheseal Thickness (mm)	Bursitis (n)	Bone Erosion (n)	Enthesophyte (n)	Entheseal Hypoecho-genicity (n)	Peri-tendinous Oedema (n)	Entheseal Vascularity (Grade)	Bursal Vascularity (Grade)
**Huang et al.** [26]	**Baseline:** 5.3 ± 0.7 **Week 2:** 0.8 ± 1.0 **Week 4:** 0.5 ± 0.6 **Week 8:** 1.1 ± 1.1 **Week 12:** 1.5 ± 0.8 *p* < 0.05 (mean ± SD)	**Baseline:** 7.6 ± 3.1 **Week 2:** 6.3 ± 2.4 **Week 4**: 6.2 ± 2.1 **Week 8:** 6.2 ± 1.9 **Week 12:** 6.2 ± 1.9 *p* > 0.05 (mean ± SD)	**Baseline:** 7 detected **Week 2:** 4 detected **Week 4**: 3 detected **Week 8:** 3 detected **Week 12:** 3 detected *p* > 0.05	**Baseline:** 4 detected **Week 2:** 4 detected **Week 4**: 4 detected **Week 8:** 4 detected **Week 12:** 4 detected *p* > 0.05	**Baseline:** 1 detected **Week 2:** 1 detected **Week 4**: 1 detected **Week 8:** 1 detected **Week 12:** 1 detected *p* > 0.05	N/A	N/A	**Baseline:** Grade 1: 0 Grade 2: 2 Grade 3: 3 Grade 4: 2 **Week 2:** Grade 1: 2 Grade 2: 4 Grade 3: 1 Grade 4: 0 **Week 4**: Grade 1: 4 Grade 2: 3 Grade 3: 0 Grade 4: 0 **Week 8:** Grade 1: 3 Grade 2: 3 Grade 3: 1 Grade 4: 0 **Week 12:** Grade 1: 2 Grade 2: 4 Grade 3: 2 Grade 4: 0	N/A
**Srivastava & Aggarwal** [27]	**Baseline:** 7 (4–10) **Week 6:** 3 (0–7) *p* < 0.0001 (mean [range])	**Baseline:** 6.9 (5.6–9.8) **Week 6:** 6.1 (4–9.8) *p* < 0.0001 (mean [range])	**Baseline:** 26 detected **Week 6:** 15 detected *p* < 0.001	**Baseline:** 24 detected **Week 6:** 24 detected *p* > 0.05	**Baseline:** 3 detected **Week 6:** 3 detected *p* > 0.05	**Baseline:** 27 detected **Week 6:** 19 detected *p* = 0.02	**Baseline:** 17 detected **Week 6:** 5 detected *p* > 0.05	**Baseline:** 2 (0–3) **Week 6:** 0 (0–3) *p* < 0.0001 (mean [grade range])	**Baseline:** 2 (0–3) **Week 6:** 0 (0–3) *p* < 0.0001 (mean [grade range])
**Level of Evidence**	Weak evidence for corticosteroid injections reducing pain as 2 studies report consistent findings	Weak evidence for corticosteroid injections decreasing entheseal thickness as 2 studies report consistent findings	Weak evidence for corticosteroid injections decreasing the presence of bursitis as 2 studies report consistent findings	Weak evidence for corticosteroid injections not improving the number of bone erosions as 2 studies report consistent findings	Weak evidence for corticosteroid injections not improving the number of enthesophytes as 2 studies report consistent findings	Inconclusive due to lack of studies	Inconclusive due to lack of studies	Weak evidence for corticosteroid injections reducing entheseal vascularity in Achilles enthesis as 2 studies report consistent findings	Inconclusive due to lack of studies

### Pain

Both studies used a visual analogue scale (VAS) as an outcome measure for pain. Neither of the two studies clarified the nominal scale used for the VAS. The pain was reported to be reduced in both observational studies [26, 27]. Huang et al. [26] only included patients that had a VAS score > 4.0 in the affected heel. The baseline VAS before betamethasone was injected was 5.3 ± 0.7 (mean ± SD), which reduced to 1.5 ± 0.8 during follow-up at week 12. However, it should be noted that the VAS reduced at week 2 (0.8 ± 1.0) and week 4 follow-up (0.5 ± 0.6) from baseline, but then increased for the next two follow-ups at week 8 and week 12. Overall, the VAS remained reduced in comparison to baseline measures [26]. Srivastava & Aggarwal found patients injected with methylprednisolone at local site of AT reported a VAS mean score of 7 with a range of 4–10 prior to injection, and a mean score of 3 with a range of 0–7 6 weeks post injection [27]. According to the evidence rating criteria by Ariens et al. [24], it can be concluded that there is weak evidence suggesting corticosteroid injections at the AT may reduce pain in the short term (6–12 weeks) as both studies reported consistent findings.

### Ultrasound evaluation (B-mode and Doppler)

Both Huang et al. [26] and Srivastava & Aggarwal [27] evaluated the Achilles enthesis and surrounding structures with B-mode US imaging and Doppler US at baseline and at follow-up visits. B-Mode US Imaging evaluated morphological changes and Doppler US assessed vascularisation at the site of AT. Both studies reported a small reduction in entheseal thickness and the presence of retrocalcaneal bursitis [26, 27]. Srivastava & Aggarwal [27] reported a reduction of entheseal thickness from 6.9 mm to 6.1 mm and a reduction in bursitis (n) from 26 at baseline to 15 at 6 week follow-up. Huang et al. [26] reported reduction of entheseal thickness from 7.6 mm to 6.2 mm and a reduction in bursitis (n) from 7 at baseline to 3 at 12 week follow-up. The values for bursitis and entheseal thickness reduced initially and remained the same from four week follow-up to twelve week follow-up [26]. No changes in the number of bone erosions and enthesophytes measured with B-mode US from baseline to follow-up were reported in both studies [26, 27]. This result is not unexpected due to the irreversible nature of these features. Srivastava & Aggarwal [27] also reported reductions in entheseal hypoechogenicity (n) from 27 to 19 and peritendinous oedema (n) from 17 to 5. Doppler US examination was undertaken in both studies to assess vascularity at the AT at baseline and follow-up. Both studies utilised different grading systems to measure vascularity (Table 5). The two studies reported a reduction in entheseal vascularity at follow-up. Huang et al. [26] reported a reduction in the number of highest grade from 2 to 0, and Srivastava & Aggarwal [27] reported a reduction in mean grade from 2 to 0. Srivastava & Aggarwal [27] also reported a reduction in retrocalcaneal bursa vascularity.

**Table 5:** – Vascularity Grading System

Vascularity Grade	Huang et al. [26]	Srivastava & Aggarwal [27]
0	N/A	No Power Doppler signals
1	No flow signal	≤3 Power Doppler signals
2	Presence of separate dot signals or short linear signals	> 3 Power Doppler signals occupying < 50% of the lesion
3	Presence of clearly discernible vascularity with either many small vessels or several long vessels with or without visible branching though involving less than half of the entheses	Power Doppler signals occupying > 50% of the lesional area
4	Severe flow signal refers to the presence of vessels involving more than half of the entheses	N/A

Overall, there is weak evidence to suggest that US guided corticosteroid injections can improve some of the B mode and Doppler US detected features at the AT in the short term (6–12 weeks). There is weak evidence to suggest an improvement in entheseal thickness (B Mode), bursitis (B Mode), and entheseal vascularity (Doppler) as both studies reported consistent findings. Weak evidence is also present to indicate no improvement in bone erosion and enthesophyte formation was observed in both studies following US guided corticosteroid injections. There is inconclusive evidence regarding entheseal hypoechogenecity (B Mode), peritendinous oedema (B Mode), and bursal vascularity (Doppler), as these features were only investigated in one study [27].

## Discussion

The aim of this study was to determine the effectiveness of non-pharmacological and corticosteroid injections in the management of the AT in people with IA. Only two studies met the inclusion criteria. These two studies investigated the effectiveness of corticosteroid injection therapy. No other relevant studies investigating the use of non-pharmacological interventions in this population were identified. A weak level of evidence was the highest possible irrespective of study quality, which in this case were low quality observational studies. To that end, weak levels of evidence were found for corticosteroid injections decreasing pain and some US (B-mode and Doppler) detected features, such as bursitis, entheseal thickness, and entheseal vascularity. All other US detectable features were either weak evidence for no improvement, which was not unexpected due to the irreversible nature of these features, or inconclusive due to a lack of studies.

The included studies, Huang et al. [26] and Srivastava & Aggarwal [27], both had low quality for internal and external validity, and thus overall low quality. The low quality ratings for internal validity were primarily attributed to the domains of selective outcome reporting/statistical issues and interventions. An inadequate sample size was present in both studies with only seven patients injected with betamethasone in Huang et al. [26], whilst 27 ATs (18 patients) were injected with methylprednisolone in Srivastava & Aggarwal [27]. Additionally, a risk of attrition bias was observed in the study by Srivastava & Aggarwal [27]. The inclusion of 40 symptomatic AT entheses (in 19 patients) was initially reported. However, only 27 symptomatic AT entheses (in 18 patients) were reported at 6-week follow-up [27]. The reason for participant drop-out was not addressed, and it is possible that some participants did not return due to adverse events or lack of improvement [28]. The interventions domain was also a concern for both studies, as the systemic management of participants was not recorded. The impact of systemic management on the effectiveness of localised pharmacologic intervention should be considered, as any localised improvements may be due to improvement in global disease activity rather than the effectiveness of the localised intervention [2]. Additionally, non-pharmacological interventions are adjunct management strategies in this population, and can have limited effect if global disease activity is not addressed [29]. Therefore, the pharmacological profile of participants with IA should always be accounted for when investigating non-pharmacological and localised pharmacological interventions.

External validity was also a focus of the quality assessment as intervention studies need to have a pragmatic approach, and be generalised to the wider population, with the aim to inform clinical practice [30]. Our findings revealed that both included studies lacked external validity. This was due to the minimal representation of females in comparison to males in both studies, with a male to female ratio of 6:1 for Huang et al. [26] and 8:1 for Srivastava & Aggarwal [27], and the lack of representation of common IA subtypes (such as RA) when IA collectively was being investigated [27]. Therefore, future intervention study design should consider population ratios for gender and prevalence of IA subtypes to allow for greater generalisability to the wider population. Whilst methodological concerns were found, both studies did demonstrate that corticosteroid injections may be an effective localised management strategy for the AT in this population. However, as the study duration from baseline to final follow-up was short (6–12 weeks), long-term efficacy or risks and adverse effects may not have been fully determined. Additionally, whilst there is evidence of decrease in pain and some radiographic features at the AT, the data reviewed did not give insight into evidence of improvement provided by corticosteroid injections in terms of disability and quality of life in people with IA.

As far as the authors are aware, this is the first systematic review to investigate the efficacy of non-pharmacological interventions and corticosteroid injections for the management of AT pathologies in people with IA. The identified studies both addressed site-specific corticosteroid injections for the management of the AT in IA. The use of corticosteroid injections for the AT is still controversial due to inconclusive evidence regarding their efficacy and potential risks [17]. This is because corticosteroid injections tend to have short-term benefits with the potential risk of weakening the structural integrity of tendons long-term. Repeated injections and possible puncture of the tendon substance can increase the potential risk of AT rupture [11]. Although evidence is limited, there are reported cases of AT rupture following corticosteroid injection in healthy population with AT pathologies [17, 31]. However, it is unknown if this is due to injection technique or the agent injected [11, 17, 32]. Notwithstanding, there is also a risk of tendon rupture in the IA population, when there is too much active inflammation at the tendon: either primary inflammation (specifically within the tendon) or secondary inflammation (from an adjacent location) [33, 34]. Therefore, the greater risk between administering an injection or not administering an injection needs to be established, and managed accordingly [17]. Consequently, the European League Against Rheumatism (EULAR) advises glucocorticosteroid injections as an adjunctive therapy for localised disease, such as enthesitis in PsA [35]. Additionally, risk of rupture due to injection technique may be mitigated by using US image guidance when injecting [36]. However, this has not been firmly proven [11]. Interestingly, both included studies administered injections under US guidance, and did not report any cases of AT rupture.

Non-pharmacological interventions for the AT, such as physical therapy consisting of eccentric exercise and ESWT, have shown positive results in the symptomatic management of the AT [16]. Additionally, non-pharmacological interventions can minimise potential risks involved with local injections, such as rupture, infection, skin hypersensitivity, and skin depigmentation [37]. However, no studies investigating non-pharmacological interventions for the management of the AT in people with IA were found during our comprehensive search. Due to the lack of evidence, there is currently no data available regarding the efficacy of non-pharmacological interventions in patients with IA. The reason is most likely due to people with IA being excluded from studies on management of the AT [17].

Exclusion may be attributed to the pathogenesis of AT pathology, which may differ between non-IA and IA, with active inflammation observed through US in patients with IA [11]. Additionally, systemic medications (such as biological drugs or DMARDs) that patients may be taking combined with a disease course of variable nature (for example, flares of disease activity and remissions) could impact the findings. If systemic management is working effectively with subsequent low disease activity, it may enhance the efficacy of results. Conversely, if systemic management is not adequately controlling disease activity, it could lead to less efficacious results for non-pharmacological or localised pharmacological interventions, and could impact adherence to interventions and study attrition rates [2]. As such, the impact of pharmacological interventions should be carefully considered in methodological design and interpretation of study outcome measures.

Therefore, due to the exclusion of people with IA from studies investigating AT management, further studies investigating non-pharmacological interventions for this population are required. Smolen et al. [38], who made recommendations for treating SpAs, also highlighted the need for more research into the management of musculoskeletal involvement. They recommended that inactive disease of musculoskeletal involvement, such as enthesitis, should be a foremost treatment target to optimise quality of life for patients. Current guidelines also highlight the importance of non-pharmacological management in overall management of axial SpA [39]. These guidelines also recommend glucocorticoid injections at the localised site of musculoskeletal inflammation could be considered to treat enthesitis despite the lack of evidence [39]. Unfortunately, guidelines with recommendations for the non-pharmacological management of IA specific conditions were not found, which further emphasises the need for more research in this area. It should also be noted that the potential mechanism of action of glucocorticoids in tendinopathy includes decreased inflammation, inhibition of cellular proliferation, scarring and adhesion, antiangiogenic activity, antinociceptive action or some combinations of these factors. The results can be positive in cases where excessive inflammation is prevalent [40, 41]. This might explain the positive results from the studies included in this systematic review due to high inflammation from the disease process of IA.

There were a number of limitations to this systematic review that need to be acknowledged. Language was limited to English due to language restrictions of the reviewers. This is generally not advised, but is difficult to overcome [42]. The number of studies that met the inclusion criteria may have been limited due to this, and the results of studies excluded based on language could have impacted on the findings of this systematic review. The second limitation of the study was that the outcome measures within each domain were the same even if the methods of determining these outcomes were different, such as reduced vascularity in the enthesis, which would be reduced regardless of the differing grading systems used by the two studies. The second assumption was that the vascularity was assumed as being decreased overall, even if it may have increased between follow-ups. The vascularity grade was still reduced at the final follow-up in comparison to the baseline grade of vascularity.

The findings of this systematic review highlight the urgent need for high quality research to be conducted to establish the efficacy of non-pharmacological interventions and injection therapies for the AT in people with IA to better guide those responsible for delivering care. Future research should consider how study outcomes may be interpreted in the context of co-interventions, such as pharmacological management, and variable disease course and progression, and consider analysis for specific subtypes of IA to allow applicability of results in wider clinical practice.

## Conclusion

There is some weak evidence for the efficacy of corticosteroid injections in reducing pain and improving some US detectable features in the AT in people with IA. The efficacy of non-pharmacological interventions could not be assessed due to a lack of relevant literature. There is an urgent need for more research in this field. Future research should address the efficacy of non-pharmacological interventions and injection therapies for the AT within the IA population, specifically addressing different subtypes of IA. An emphasis should also be placed on external validity to allow for greater applicability in clinical practice.

## Supplementary Information

**Additional File 1:.** Search Strategies for non-pharmacological interventions and corticosteroid injections for the management of the Achilles tendon in inflammatory arthritis: A systematic review

## Data Availability

Data sharing is not applicable to this article as no datasets were generated or analysed during the current study.

## Abbreviations

AT: Achilles tendon
AS: Ankylosing spondylitis
DMARDs: Disease modifying anti-rheumatic drugs
EULAR: European league against rheumatism
ESWT: Extracorpeal shockwave therapy
IA: Inflammatory arthritis
NSAIDs: Non-steroidal anti-inflammatory drugs
PsA: Psoriatic arthritis
RA: Rheumatoid arthritis
RCT: Randomised controlled trial
SpA: Spondyloarthropathy
US: Ultrasound
VAS: Visual analogue scale
