# Supplementary material for: Non-pharmacological interventions and corticosteroid injections for the management of the Achilles tendon in inflammatory arthritis: a systematic review
Source: J Foot Ankle Res. 2021 Jul 10;14:48. doi: 10.1186/s13047-021-00484-6 (PMC8272330; doi:10.1186/s13047-021-00484-6)
Supplement: Supplementary file 1 — Additional File 1:. Search Strategies for non-pharmacological interventions and corticosteroid injections for the management of the Achilles tendon in inflammatory arthritis: A systematic review [file 13047_2021_484_MOESM1_ESM.docx]

**Additional File 1: Search Strategies for non-pharmacological interventions and corticosteroid injections for the management of the Achilles tendon in inflammatory arthritis: A systematic review**

Database search conducted on August 3^rd^, 2020.

*Medline Search Strategy*

1. MH “Achilles Tendon”
2. achilles
3. enthes*
4. 1 **OR** 2 **OR** 3
5. MH “Therapeutics”
6. treatment
7. management
8. intervention
9. therap*
10. non-pharmacological
11. conservative
12. MH “Surgical Procedures, Operative”
13. surg*
14. MH “Injections”
15. MH “Physical Therapy Modalities”
16. MH “Motion Therapy, Continuous Passive”
17. MH “Musculoskeletal Manipulations”
18. MH “Orthotic Devices”
19. MH “Splints”
20. MH “Casts, Surgical”
21. MH “Foot Orthoses”
22. orthos*
23. insole
24. “joint protection”
25. MH “Shoes”
26. footwear
27. MH “Complementary Therapies”
28. MH “Exercise”
29. MH “Resistance Training”
30. “strength training”
31. “physical training”
32. eccentric
33. concentric
34. isometric
35. electrophysical
36. MH “Ultrasonic Therapy”
37. MH “Extracorporeal Shockwave Therapy”
38. MH “Hydrotherapy”
39. “laser therapy”
40. thermotherapy
41. MH “Diathermy”
42. MH “Cryotherapy”
43. MH “Acupuncture Therapy”
44. “dry needling”
45. MH “Patient Education as Topic”
46. 5 **OR** 6 **OR** 7 **OR** 8 **OR** 9 **OR** 10 **OR** 11 **OR** 12 **OR** 13 **OR** 14 **OR** 15 **OR** 16 **OR** 17 **OR** 18 **OR** 19 **OR** 20 **OR** 21 **OR** 22 **OR** 23 **OR** 24 **OR** 25 **OR** 26 **OR** 27 **OR** 28 **OR** 29 **OR** 20 **OR** 31 **OR** 32 **OR** 33 **OR** 34 **OR** 35 **OR** 36 **OR** 37 **OR** 38 **OR** 39 **OR** 40 **OR** 41 **OR** 42 **OR** 43 **OR** 44 **OR** 45
47. 4 **AND** 46

*Embase Search Strategy*

1. achilles tendon/
2. achilles.mp
3. enthes*.mp
4. 1 **OR** 2 **OR** 3
5. therapy/
6. treatment.mp
7. management.mp
8. intervention.mp
9. therap*.mp
10. non-pharmacological.mp
11. conservative treatment/
12. surgery/
13. surg*.mp
14. injection/
15. physical therapy modalities/
16. musculoskeletal Manipulation/
17. movement therapy/
18. orthosis/
19. splint/
20. orthopedic cast/
21. foot orthosis/
22. orthos*.mp
23. insole.mp
24. joint protection.mp
25. shoe/
26. footwear.mp
27. complementary therapies.mp
28. exercise/
29. resistance training/
30. strength training.mp
31. physical training.mp
32. eccentric.mp
33. concentric.mp
34. isometric.mp
35. electrophysical.mp
36. ultrasound therapy/
37. shock wave therapy/
38. hydrotherapy/
39. low level laser therapy/
40. thermotherapy/
41. diathermy/
42. cryotherapy/
43. acupuncture/
44. dry needling.mp
45. patient education/
46. 5 **OR** 6 **OR** 7 **OR** 8 **OR** 9 **OR** 10 **OR** 11 **OR** 12 **OR** 13 **OR** 14 **OR** 15 **OR** 16 **OR** 17 **OR** 18 **OR** 19 **OR** 20 **OR** 21 **OR** 22 **OR** 23 **OR** 24 **OR** 25 **OR** 26 **OR** 27 **OR** 28 **OR** 29 **OR** 20 **OR** 31 **OR** 32 **OR** 33 **OR** 34 **OR** 35 **OR** 36 **OR** 37 **OR** 38 **OR** 39 **OR** 40 **OR** 41 **OR** 42 **OR** 43 **OR** 44 **OR** 45
47. 4 **AND** 46

*CINAHL Search Strategy*

1. MH “Achilles Tendon”
2. achilles
3. enthes*
4. 1 **OR** 2 **OR** 3
5. MH “Therapeutics”
6. treatment
7. management
8. intervention
9. therap*
10. non-pharmacological
11. conservative
12. MH “Surgical Procedures, Operative”
13. surg*
14. MH “Injections”
15. MH “Physical Therapy Modalities”
16. MH “Motion Therapy, Continuous Passive”
17. MH “Musculoskeletal Manipulations”
18. MH “Orthotic Devices”
19. MH “Splints”
20. MH “Casts, Surgical”
21. MH “Foot Orthoses”
22. orthos*
23. insole
24. “joint protection”
25. MH “Shoes”
26. footwear
27. MH “Complementary Therapies”
28. MH “Exercise”
29. MH “Resistance Training”
30. “strength training”
31. “physical training”
32. eccentric
33. concentric
34. isometric
35. electrophysical
36. MH “Ultrasonic Therapy”
37. MH “Extracorporeal Shockwave Therapy”
38. MH “Hydrotherapy”
39. “laser therapy”
40. thermotherapy
41. MH “Diathermy”
42. MH “Cryotherapy”
43. MH “Acupuncture Therapy”
44. “dry needling”
45. MH “Patient Education as Topic”
46. 5 **OR** 6 **OR** 7 **OR** 8 **OR** 9 **OR** 10 **OR** 11 **OR** 12 **OR** 13 **OR** 14 **OR** 15 **OR** 16 **OR** 17 **OR** 18 **OR** 19 **OR** 20 **OR** 21 **OR** 22 **OR** 23 **OR** 24 **OR** 25 **OR** 26 **OR** 27 **OR** 28 **OR** 29 **OR** 20 **OR** 31 **OR** 32 **OR** 33 **OR** 34 **OR** 35 **OR** 36 **OR** 37 **OR** 38 **OR** 39 **OR** 40 **OR** 41 **OR** 42 **OR** 43 **OR** 44 **OR** 45
47. 4 **AND** 46

*Cochrane Library Search Strategy*

1. MeSH Descriptor: [Achilles Tendon]
2. achilles
3. enthes*
4. 1 **OR** 2 **OR** 3
5. MeSH Descriptor: [Therapeutics]
6. treatment
7. management
8. intervention
9. therap*
10. non-pharmacological
11. conservative
12. MeSH Descriptor: [Surgical Procedures, Operative]
13. surg*
14. MeSH Descriptor: [Injections]
15. MeSH Descriptor: [Physical Therapy Modalities]
16. MeSH Descriptor: [Motion Therapy, Continuous Passive]
17. MeSH Descriptor: [Musculoskeletal Manipulations]
18. MeSH Descriptor: [Orthotic Devices]
19. MeSH Descriptor: [Splints]
20. MeSH Descriptor: [Casts, Surgical]
21. MeSH Descriptor: [Foot Orthoses]
22. orthos*
23. insole
24. “joint protection”
25. MeSH Descriptor: [Shoes]
26. footwear
27. MeSH Descriptor: [Complementary Therapies]
28. MeSH Descriptor: [Exercise]
29. MeSH Descriptor: [Resistance Training]
30. “strength training”
31. “physical training”
32. eccentric
33. concentric
34. isometric
35. electrophysical
36. MeSH Descriptor: [Ultrasonic Therapy]
37. MeSH Descriptor: [Extracorporeal Shockwave Therapy]
38. MeSH Descriptor: [Hydrotherapy]
39. “laser therapy”
40. thermotherapy
41. MeSH Descriptor: [Diathermy]
42. MeSH Descriptor: [Cryotherapy]
43. MeSH Descriptor: [Acupuncture Therapy]
44. “dry needling”
45. MeSH Descriptor: [Patient Education as Topic]
46. 5 **OR** 6 **OR** 7 **OR** 8 **OR** 9 **OR** 10 **OR** 11 **OR** 12 **OR** 13 **OR** 14 **OR** 15 **OR** 16 **OR** 17 **OR** 18 **OR** 19 **OR** 20 **OR** 21 **OR** 22 **OR** 23 **OR** 24 **OR** 25 **OR** 26 **OR** 27 **OR** 28 **OR** 29 **OR** 20 **OR** 31 **OR** 32 **OR** 33 **OR** 34 **OR** 35 **OR** 36 **OR** 37 **OR** 38 **OR** 39 **OR** 40 **OR** 41 **OR** 42 **OR** 43 **OR** 44 **OR** 45
47. 4 **AND** 46
